# The Role of Race and Ethnicity on Time to Treatment in Orthopaedic Oncology

**DOI:** 10.3390/cancers18061006

**Published:** 2026-03-20

**Authors:** Melissa Romoff, Michael S. Kim, Madison Brunette, Mitchell S. Fourman, Russell Stitzlein, Amanda N. Goldin

**Affiliations:** 1 Department of Orthopaedic Surgery, University of California Irvine, Orange, CA 92868, USA; mromoff@hs.uci.edu (M.R.); michak14@hs.uci.edu (M.S.K.); brunettm@hs.uci.edu (M.B.); rstitzle@hs.uci.edu (R.S.); 2 Department of Orthopaedic Surgery, Montefiore Einstein, Bronx, NY 10467, USA; mfourman@montefiore.org

**Keywords:** orthopaedic oncology, time to treatment, health disparities, sarcoma, surgical delay

## Abstract

**Background:** Timely treatment is critical for patients with bone and soft tissue tumors, but access to care may not be equitable across all populations. While treatment delays have been well studied in other cancers, disparities in time to treatment remain underexplored in orthopaedic oncology. This study aimed to determine whether racial or ethnic disparities exist in the timing of surgery, chemotherapy, or radiation for patients with sarcoma or metastatic bone disease. **Methods:** A retrospective cohort study was conducted using the TriNetX US Collaborative Network, a multi-institutional electronic health record database. Adult patients undergoing biopsy and subsequently diagnosed with bone sarcoma, soft tissue sarcoma, or metastatic bone disease were identified. Time to treatment was defined as the number of days between biopsy and the first recorded surgery, chemotherapy, or radiation. Patients were stratified by race and ethnicity, and statistical comparisons were performed using Mann–Whitney U tests and t-tests. **Results:** A total of 63,087 patients met inclusion criteria (55,697 with metastatic bone disease/bone sarcoma and 7390 with soft tissue sarcoma). In the metastatic/bone sarcoma cohort, Hispanic patients had shorter mean time to resection (58 ± 94 vs. 82 ± 239 days, *p* = 0.008) and fixation (35 ± 142 vs. 72 ± 315 days, *p* < 0.001) compared to non-Hispanic patients, although median times did not differ significantly. Among black patients, time to fixation was shorter than in White patients (mean 22 ± 103 vs. 114 ± 468 days, *p* < 0.001; median 0 days in both groups), while delays were observed in time to radiation (median 13 vs. 7 days; mean 85 ± 284 vs. 43 ± 203 days, *p* < 0.001). In the soft tissue sarcoma cohort, Black patients experienced longer mean times to resection (142 ± 293 vs. 79 ± 216 days) and radiation (141 ± 514 vs. 96 ± 364 days), though comparisons were limited by sample size. **Conclusions:** This large, multi-institutional study demonstrates that disparities in orthopaedic oncology differ by treatment modality and clinical context. Shorter wait times to surgery among Hispanic and Black patients in metastatic disease likely reflect more advanced disease presentation and barriers to early access, whereas delays in resection and radiation highlight inequities in accessing non-emergent, coordinated oncologic care. Reporting both means and medians provides a more complex understanding of treatment delays and underscores the need for interventions that expand early access to orthopaedic oncologists and ensure timely, equitable care.

## Simple Summary

Delays in cancer treatment can lead to worse outcomes, yet limited data exists on how these delays affect patients with bone and soft tissue malignancies. Orthopaedic oncologists specialize in managing both benign and malignant bone and soft tissue tumors, including sarcomas, which are rare mesenchymal cancers requiring surgery, often alongside chemotherapy and/or radiation. Orthopaedic oncologists also surgically manage metastatic cancer to bone when fractures are impending or have occurred. Unfortunately, delays in diagnosis and treatment are common. Using a large national database, this study examined whether time to treatment differs by race and ethnicity in patients with bone sarcomas, soft tissue sarcomas, and metastatic cancer to bone. We found that Hispanic and Black patients often underwent surgery sooner in the metastatic setting, likely reflecting presentation with more advanced disease, such as displaced pathologic fracture. However, both groups experienced longer delays in non-emergent treatments, including resection and radiation when compared to white patients. These findings highlight the importance of improving early access to orthopaedic oncologists and developing care pathways that ensure all patients receive timely, coordinated cancer treatments.

## 1. Introduction

Orthopaedic oncologists care for a heterogenous group of patients with primary bone and soft tissue sarcomas as well as metastatic bone disease, a disease spectrum that includes both rare malignancies and a common complication of advanced solid tumors. These disease processes require timely multidisciplinary management, often including surgery and chemotherapy, and/or radiation to prevent the local progression and metastasis of primary tumors [1,2,3]. Variability in tumor growth patterns [4,5], early metastatic potential [6], and frequent delays in diagnosis [7,8,9,10,11] contribute to a poor prognosis and mortality. Diagnostic delays are frequently driven by vague early symptoms [12,13] and limited sarcoma exposure in the primary care setting [14,15], hindering timely referral and specialist evaluation. Recent initiatives have aimed to improve the early recognition of sarcoma and referral for specialist care through education on the warning signs of this disease [16,17]. After diagnosis, delays in the initiation of definitive treatment can significantly compromise oncologic outcomes, making time to treatment a critical yet under-explored determinant of survival.

Disparities in time to treatment have been well documented across oncologic subspecialties. In U.S.-based studies, Black and Hispanic patients with breast and colorectal cancers experience longer delays from diagnosis to surgery and adjuvant therapies, while comparable delays have been observed in international cohorts, including studies from Australia [18,19]. A large meta-analysis found that even a one-month delay in cancer treatment significantly increased mortality [19]. Surgery delays were associated with a 6–8% higher risk of death, and a 12-week delay in breast cancer surgery increased mortality by 28% [19]. These treatment delays are well recognized drivers of preventable complications and reflect structural inequities in healthcare access.

Despite the time-sensitive nature of metastatic bone disease and sarcoma care, no large-scale, multi-institutional studies have evaluated whether such disparities exist in orthopaedic oncology. This is concerning given the complexity of these patients, which requires coordination across multiple medical specialties. The objective of this study is to determine whether race and ethnicity are associated with disparities in time to treatment among patients with bone or soft tissue sarcomas and skeletal metastatic disease using a national, multi-institutional database.

## 2. Materials and Methods

### 2.1. Study Design and Database

This study was a retrospective cohort analysis utilizing the TriNetX US Collaborative Network, a federated, multi-institutional research platform that aggregates de-identified electronic health record data from participating healthcare organizations across the United States. The network includes academic medical centers, community hospitals, and integrated health systems, collectively representing over 117 million patients. TriNetX adheres to HIPAA regulations for data security and confidentiality, and all datasets are fully de-identified, making this study exempt from IRB approval.

### 2.2. Cohort Selection

Eligible patients were identified retrospectively from the TriNetX US Collaborative Network using electronic health record data queried between 2014 and 2023. Adult patients (≥18 years) who underwent a tissue biopsy (needle or open) were identified using CPT codes (Supplementary Table S1). From this group, three initial diagnostic cohorts were defined based on a new diagnosis of bone sarcoma, soft tissue sarcoma, or metastatic bone disease, using ICD-10 codes (Supplementary Table S1). To ensure adequate capture of post-biopsy treatment initiation, inclusion was limited to patients with at least two years of documented follow-up after the index biopsy.

During the preliminary analyses, unexpectedly high rates of radiation use in the bone sarcoma cohort, including cases where radiation appeared as the first line treatment were observed. Because this pattern was inconsistent with clinical practice, and likely reflected miscoding of metastatic disease to bone under C40-41 ICD-10 codes, patients with bone sarcoma and metastatic bone disease were combined into a single analytic cohort. This combined approach was chosen to avoid misleading treatment patterns and to ensure more clinically reliable results.

Within the soft tissue sarcoma and combined bone/metastatic cohorts, we identified the proportion of patients who subsequently underwent surgical resection, chemotherapy, and/or radiation therapy using CPT codes and TriNetX-curated procedural definitions. Time to treatment was calculated as the number of days from biopsy to the first recorded date of corresponding treatment intervention.

For the combined bone/metastatic cohort, patients who underwent surgical intervention using an expanded set of CPT codes capturing a broad range of operative fixation procedures were identified. This approach was designed to include both prophylactic fixation (e.g., intramedullary nailing for impending fracture) and post-fracture surgical fixation (e.g., displaced pathologic fractures), to better reflect real-world presentation patterns and surgical urgency (Supplementary Table S1). In addition to analyzing time to surgery across the entire surgical cohort, subgroup analysis restricted to patients who underwent prophylactic fixation procedures only were performed, to evaluate whether racial disparities in time to treatment were more pronounced in non-urgent settings.

### 2.3. Exposure and Stratification

Patients were stratified by race (White, Black, Asian, Other) and ethnicity (Hispanic vs. Non-Hispanic) based on self-reported demographic data within TriNetX.

### 2.4. Statistical Analysis

All patient counts, demographic distributions, and time-to-treatment data were generated using the TriNetX analytics platform. These results are summarized in Table 1, Table 2, Table 3 and Table 4 using descriptive statistics, including both means and medians, along with standard deviations to reflect the substantial skew and variability across patient cohorts. To evaluate differences in treatment timing between racial and ethnic groups, group comparisons using appropriate statistical tests in GraphPad Prism (11.0.0) (La Jolla, CA, USA) were conducted, which are presented in Table 1, Table 2, Table 3 and Table 4. Means were compared using independent-samples t-tests, while medians were compared using the Mann–Whitney U test given the skewed distribution of the data. These analyses were limited to groups with sufficient sample sizes to allow for meaningful interpretation. Patient numbers in the Asian and Other categories were too small to permit reliable statistical analyses.

Given the highly skewed and zero-inflated distribution of time-to-treatment data, both parametric (Welch’s *t*-test) and nonparametric (Mann–Whitney U) tests were used to compare group differences. These tests were intentionally applied in parallel to evaluate differences in both mean treatment delays, which capture the burden of extreme delays, and median treatment timing, which reflects the typical patient experience.

## 3. Results

A total of 63,087 patients that met the inclusion criteria were stratified into two cohorts based on diagnosis (Table 1, Table 2, Table 3 and Table 4): metastatic bone disease/bone sarcoma (*n* = 55,697), and soft tissue sarcoma (*n* = 7390). Patients were further stratified by ethnicity (Hispanic vs. Non-Hispanic) and race (White, Black, Asian, Other,) and time from biopsy to first recorded treatment (surgical resection, prophylactic or fracture fixation, chemotherapy, or radiation) was assessed (Table 1, Table 2, Table 3 and Table 4).

### 3.1. Metastatic/Bone Sarcoma Cohort

Among patients in the metastatic/bone sarcoma cohort, time-to-treatment distributions were highly skewed, as evidenced by means that were consistently much greater than corresponding medians (Table 1, Table 2, Table 3 and Table 4). Median time to surgery and prophylactic fixation was zero across all racial and ethnic groups (Table 1 and Table 3). This finding indicates that at least 50% of patients underwent operative intervention on the same day as, or within a few days of, biopsy. The distribution of surgical timing is therefore heavily right-skewed: the majority of patients had near immediate surgery, while a smaller, yet significant, subset experienced substantial delays, which elevated the mean values. Reporting both medians and means captures the pattern more accurately than either measure alone.

**Figure 1:**
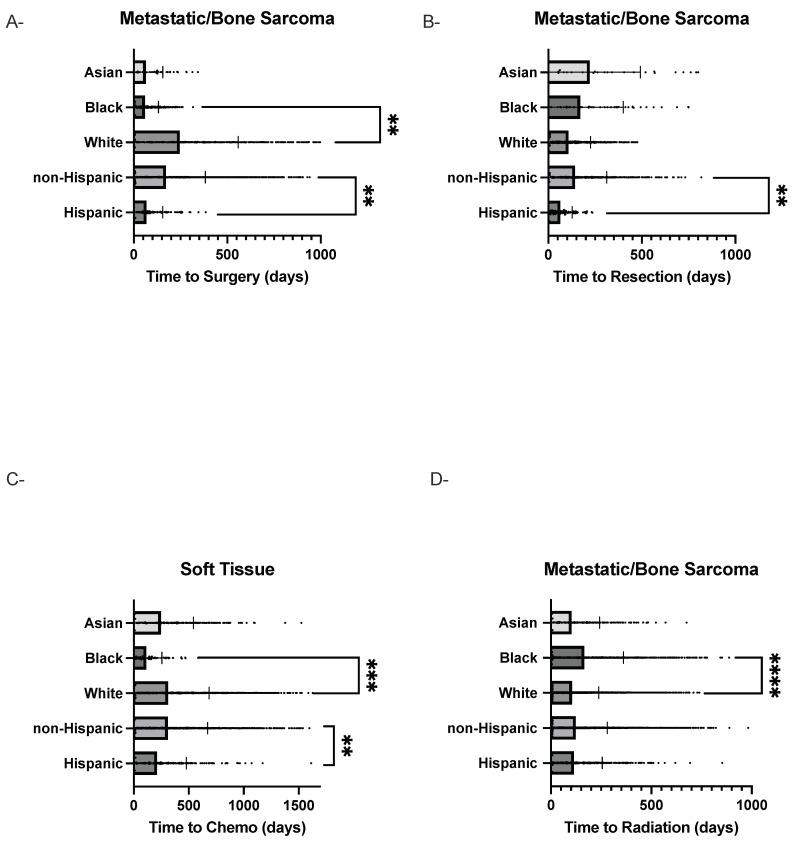
Mean Time to Treatment by Race and Ethnicity. Scatter box plots illustrate the distribution of time (days) from biopsy to treatment initiation across racial and ethnic groups. Individual points represent patients, while boxes display the interquartile range with the mean indicated by the central marker. (**A**) Time to surgery in patients with metastatic bone disease/bone sarcoma. (**B**) Time to surgical resection in the metastatic bone sarcoma cohort. (**C**) Time to chemotherapy in patients with soft tissue sarcoma. (**D**) Time to radiation in the metastatic/bone sarcoma cohort. Groups are stratified by race (Asian, Black, White) and ethnicity (Hispanic vs. non-Hispanic). Sample sizes for each treatment cohort were surgery (*n* = 2355), resection (*n* = 846), chemotherapy (*n* = 2062), and radiation (*n* = 7208). Asterisks indicate statistically significant differences between groups (** *p* < 0.01, *** *p* < 0.001, **** *p* < 0.0001).

Among Hispanic and non-Hispanic patients, significant differences were observed in time to treatment. Hispanic patients had a shorter mean time to resection (58 ± 94 vs. 82 ± 239, *p* = 0.008) and shorter mean time to surgery (35 ± 142 vs. 72 ± 315, *p* < 0.001) (Table 2, Figure 1A–D). Median comparisons using Mann–Whitney U tests showed no statistically significant differences (Table 1).

Among Black and White patients, significant delays were observed in radiation timing for Black patients (median 13 (9–166) vs. 7 (4–98) days, *p* < 0.001; mean 85 ± 284 vs. 43 ± 203 days, *p* < 0.001) (Table 3 and Table 4). Time to surgery was shorter among Black patients compared to White patients (mean 22 ± 103 vs. 114 ± 468 days, 0.001).

### 3.2. Soft Tissue Sarcoma Cohort

Among Hispanic and non-Hispanic patients, mean time to chemotherapy was significantly shorter for Hispanic patients (114 ± 389 vs. 160 ± 521, *p* = 0.001). No significant differences were observed in time to radiation or surgical resection between these groups.

In the Black versus White comparison, White patients had significantly longer mean time to chemotherapy (160 ± 521 vs. 128 ± 389, *p* = 0.002). For radiation, Black patients trended towards longer mean and median times compared to White patients (141 ± 514 vs. 96 ± 364; medians 29 (17–310) vs. 19 (13–191) days) Similarly, Black patients also trended towards longer mean and median times to surgical resection (142 ± 293 vs. 79 ± 216; median 36 (22–188) vs. 28 (19–113) days). Interpretation of radiation and resection differences was limited by sample size.

Overall, 63,087 patients met inclusion criteria (55,697 metastatic/bone sarcoma; 7390 soft tissue sarcoma).

## 4. Discussion

In this large, multi-institutional cohort, disparities in time to treatment were identified by both ethnicity and race across patients with primary bone sarcoma/metastatic bone disease and soft tissue sarcoma.

Notably, the pattern observed in orthopaedic oncology differs from many solid-organ cancers in that minority patients often underwent surgical fixation sooner in the metastatic setting. This contrast likely reflects disease severity at presentation rather than improved access, as urgent intervention for displaced pathologic fractures or neurologic compromise proceed promptly once patients enter the healthcare system. In this respect, orthopaedic oncology shares features with other time-sensitive surgical fields, such as trauma and emergency general surgery, where disparities are more pronounced upstream in access and referral rather than in emergent care delivery itself. These similarities and differences highlight that disparities in musculoskeletal oncology are driven less by delays once patients present for urgent care and more by systemic barriers to early diagnosis, specialty referral, and coordinated multidisciplinary treatment.

Across both diagnostic groups, treatment timing distributions were highly skewed, as evidenced by large differences between the mean and median values. In the metastatic/bone sarcoma cohort, median times to surgery and prophylactic fixations were zero across all groups, indicating that at least half of patients underwent operative intervention at the time of biopsy or shortly thereafter. This pattern likely reflects the clinical reality that many patients present with an urgent indication for fixation, such as a displaced pathologic fracture, necessitating immediate treatment, and/or biopsy at the same time as the operative stabilization. However, the long right tail of the distribution in both cohorts demonstrates that a meaningful, and often significant, subset of patients experienced substantial delays, elevating mean values and underscoring the importance of reporting both mean and median times. In this context, the mean provides additional clinical insight by capturing the range and severity of delays, whereas the median alone risks obscuring disparities in patients who waited longest for care.

The observed patterns likely reflect structural or systemic barriers that limit timely access to musculoskeletal oncology care for both Hispanic and Black patients. Among Hispanic patients, disparities were most evident in the metastatic/bone sarcoma cohort. Hispanic patients demonstrated shorter mean times to surgical resection and fixation, a finding that likely reflects presentation with more advanced disease requiring urgent intervention, such as displaced pathologic fracture. This pattern suggests a bottleneck in accessing musculoskeletal oncology care early in the disease course, where timely care may have enabled prophylactic fixation or elective resection before urgent surgery became necessary. In contrast, when examining other treatment modalities such as chemotherapy and radiation, no consistent delays were observed. This divergence underscores that disparities appear concentrated in the surgical domain, likely reflecting the limited number of orthopaedic oncologists nationally and the challenges patients face in reaching these specialists before disease progression. Prior studies in breast, colorectal, and gynecologic cancers have shown that Hispanic patients face socioeconomic constraints, limited availability of specialty providers, and differences in referral timing as contributors to delayed treatment [20,21,22,23]. Cultural and historical factors may shape engagement with care, with research demonstrating heightened medical mistrust in some Hispanic subpopulations, particularly recent immigrants from regions where healthcare systems have been perceived as exploitative or inconsistent in quality [24,25,26].

Racial disparities revealed a similar but distinct pattern. In the metastatic/bone sarcoma cohort, Black patients had shorter mean times to surgical fixation compared to White patients, again likely reflecting urgent presentation rather than improved access. Outside of emergent settings, delays were more commonly observed. Black patients with metastatic disease experienced longer mean and median times to radiation and surgical resection. Unlike fixation, these modalities are typically planned in an outpatient setting, suggesting systemic barriers such as referral delays, scheduling challenges, or access to oncology infrastructure. Similarly, extensive literature across breast, colorectal, and prostate cancers has documented that Black patients are more likely to encounter delays in referral, reduced access to high-volume specialty centers, and barriers related to insurance coverage [27,28,29]. Within musculoskeletal oncology, these challenges are magnified by limited number of orthopaedic oncologists—a subspeciality with fewer than 300 providers nationwide, largely concentrated at academic centers [30,31].

This study is subject to limitations inherent to those of a retrospective database analysis. TriNetX does not capture tumor-specific characteristics such as size, grade, or stage, as well as insurance status or referral pathways that likely influence treatment timing. While sample sizes were large, stratification reduced cohort sizes in some subgroups, limiting the generalizability. Furthermore, race and ethnicity in TriNetX are based on self-reported data at the point of care. There is no universal definition for what qualifies as “Hispanic,” and patients who self-identify as Hispanic may differ widely in language, country of origin, and cultural background. Although often treated as uniform categories in clinical research, race and ethnicity are complex, context-dependent constructs. These limitations are important to consider when interpreting subgroup differences.

To address coding overlap and potential misclassification, patients with bone sarcoma and metastatic bone disease were combined into a single cohort; while this approach reduced the risk of double-counting and improved internal consistency, it may have obscured nuances in treatment patterns unique to primary versus metastatic disease. Furthermore, TriNetX cannot reliably assess treatment sequencing, which is a critical consideration in sarcoma management where neoadjuvant therapy is often the first-line approach. Inability to determine whether radiation or chemotherapy preceded surgery may obscure subtle disparities in care coordination. Future studies incorporating clinical records and sequencing data are needed to fully elucidate mechanisms contributing to observed disparities. Finally, while the registry-based approach to this study can only assess treatment delays in quantitative terms, it cannot capture the broader contextual drivers of inequity. Taken together, socioeconomic disadvantage, referral delays, limited specialty availability and medical mistrust represent systemic forces that likely underlie the disparities we observed.

## 5. Conclusions

This study represents one of the first large-scale, multi-institutional analyses to evaluate racial and ethnic disparities in time to treatment among orthopaedic oncology patients. It was observed that Hispanic and Black patients often underwent fixation or resection sooner in the metastatic setting, a pattern likely reflecting presentation with more advanced disease requiring urgent surgical intervention. In contrast, both groups experienced longer delays in non-emergent, coordinated oncologic treatment, resection among Hispanic patients and radiation and resection among Black patients. These findings underscore that disparities in musculoskeletal oncology are shaped less by emergent interventions, which proceed promptly once patients present, and more upstream barriers in early referral and coordinated care. Importantly, the wide gap between mean and median times demonstrates that average delays, not just typical patient experience, capture the true burden of inequity. Future work should incorporate tumor-specific, socioeconomic, and insurance-related factors to clarify the drivers of delay and inform strategies that expand early access to orthopaedic oncologists and promote timely, equitable care.

## Figures and Tables

**Table 1:** Median Time to Treatment by Ethnicity in Sarcoma and Metastatic Cancer Cohorts.

Diagnostic Group	Treatment Type	Hispanic (*n*)	Non-Hispanic (*n*)	Median (IQR) (Hispanic)	Median (IQR) (Non-Hispanic)	*p*-Value
Metastatic/Bone Sarcoma	Chemo	1278	12,785	15 (0–72)	16 (0–91)	0.722
	Radiation	506	6702	13 (3–82)	12 (2–94)	0.630
	Resection	110	736	35 (15–77)	28 (9–94)	0.428
	Surgery	166	2189	0 (0–58)	0 (0–91)	0.378
	Prophylactic	216	2301	0 (0–115)	0 (0–92)	0.236
Soft Tissue	Chemo	266	1796	17 (9–129)	19 (12–215)	0.305
	Radiation	94	784	17 (11–172)	19 (12–131)	0.535
	Resection	79	603	35 (19–102)	28 (13–143)	0.098

Median time (in days) from biopsy to treatment are shown for Hispanic and non-Hispanic patients with metastatic cancer/bone sarcoma and soft tissue sarcoma. *p*-values reflect results from Mann–Whitney U tests comparing treatment timing by ethnicity.

**Table 2:** Mean Time to Treatment by Ethnicity Across Sarcoma and Metastatic Cancer Cohorts.

Diagnostic Group	Treatment Type	Hispanic (*n*)	Non-Hispanic (*n*)	Mean ± SD (Hispanic)	Mean ± SD (Non-Hispanic)	*p*-Value
Metastatic/Bone Sarcoma	Chemo	1278	12,785	112 ± 332	105 ± 361	0.2194
	Radiation	506	6702	59 ± 206	56 ± 236	0.7854
	Resection	110	736	58 ± 94	82 ± 239	0.008
	Surgery	166	2189	35 ± 142	72 ± 315	<0.001
	Prophylactic	216	2301	86 ± 454	82 ± 387	0.9308
Soft Tissue	Chemo	266	1796	114 ± 389	160 ± 521	0.0010
	Radiation	94	784	100 ± 301	100 ± 392	0.2945
	Resection	79	603	73 ± 143	85 ± 229	0.1581

Mean (± SD) time (in days) from biopsy to treatment are shown for Hispanic and non-Hispanic patients with metastatic cancer/bone sarcoma and soft tissue sarcoma. *p*-values reflect results comparisons of group means using two-sample *t*-tests (Welch’s correction applied for unequal variance).

**Table 3:** Median Time to Treatment by Race in Sarcoma and Metastatic Cancer Cohorts.

Diagnostic Group	Treatment Type	Black (*n*)	White (*n*)	Median (Black)	Median (White)	*p*-Value
Metastatic/Bone Sarcoma	Chemo	2259	12,987	17 (5–193)	13 (8–154)	0.333
	Radiation	1176	6657	13 (9–166)	7 (4–98)	<0.001
	Resection	81	673	21 (11–184)	21 (10–92)	0.652
	Surgery	327	748	0 (0–43)	0 (0–76)	0.671
	Prophylactic	347	2380	0 (0–102)	0 (0–172)	0.496
Soft Tissue	Chemo	295	1757	21 (13–211)	21 (10–392)	0.256
	Radiation	138	749	29 (17–310)	19 (13–191)	0.189
	Resection	86	597	36 (22–188)	28 (19–113)	0.059

Median time (in days) from biopsy to treatment are shown for Black and White patients with metastatic cancer/bone sarcoma and soft tissue sarcoma. *p*-values reflect results from Mann–Whitney U tests comparing treatment timing by ethnicity.

**Table 4:** Mean Time to Treatment by Race in Sarcoma and Metastatic Cancer Cohorts.

Diagnostic Group	Treatment Type	Black (*n*)	White (n)	Mean ± SD (Black)	Mean ± SD (White)	*p*-Value
Metastatic/Bone Sarcoma	Chemo	2259	12,987	99 ± 306	109 ± 347	0.434
	Radiation	1176	6657	85 ± 284	43 ± 203	<0.001
	Resection	81	673	75 ± 327	55 ± 162	0.6251
	Surgery	327	748	22 ± 103	114 ± 468	<0.001
	Prophylactic	347	2380	56 ± 276	84 ± 397	0.1566
Soft Tissue	Chemo	295	1757	143 ± 438	167 ± 542	0.0005
	Radiation	138	749	141 ± 514	96 ± 364	0.4949
	Resection	86	597	142 ± 293	79 ± 216	0.1907

Mean time (in days) from biopsy to treatment are shown for Black and White patients with metastatic cancer/bone sarcoma and soft tissue sarcoma. *p*-values reflect results comparisons of group means using two-sample *t*-tests (Welch’s correction applied for unequal variance).

## Data Availability

The data used in this study was obtained from the TriNetX US Collaborative Network. Due to patient privacy protections and data use agreements, the underlying individual-level data cannot be shared publicly. Aggregate-level data are available within the TriNetX platform to authorized users.

## Supplementary Materials

The following supporting information can be downloaded at: https://www.mdpi.com/article/10.3390/cancers18061006/s1, Supplementary Table S1. ICD/CPT/TriNetX Curated codes.
