# Supplementary material for: The Role of Race and Ethnicity on Time to Treatment in Orthopaedic Oncology
Source: Cancers (Basel). 2026 Mar 20;18(6):1006. doi: 10.3390/cancers18061006 (PMC13025552; doi:10.3390/cancers18061006)
Supplement: Supplementary file 1 [file cancers-18-01006-s001.zip › cancers-4107097-supplementary.pdf]

Supplementary Table 1. ICD/CPT/TriNetX Curated codes

Bone Biopsy:
20225, 20220, 20240, 20245

Soft Tissue Biopsy:
21920, 21925, 23065, 23066, 24065, 24066, 25065, 25066, 27040, 27041, 27323, 27324, 27613, 27614

Bone Sarcoma:
C40, C41

Soft Tissue Sarcoma:
C49

Metastatic Bone Disease
C79.5

Bone Resection:
23195, 23200, 23220, 24150, 24152, 25170, 26250, 26260, 26262, 27075, 27076, 27077, 27078, 27080, 27365, 27645, 27646, 27647, 28171, 28173, 28175

Soft Tissue Resection:
21925, 21936, 23077, 23078, 24077, 24079, 25077, 25078, 27049, 27059, 27329, 27364, 27615, 27616, 28046, 28047

Chemotherapy (TriNetX Curated):
1002

Radiation (TriNetX Curated):
1010843

Surgery:
23515, 23615, 23616, 24515, 24516, 24545, 24546, 24566, 24575, 24579, 24582, 24586, 24587, 24665, 24666, 24685, 25515, 25545, 25574, 25575, 25607, 25608, 25609, 25628, 25645, 25651, 25652, 26608, 26615, 26735, 26746, 26765, 27215, 27216, 27217, 27218, 27226, 27227, 27228, 27235, 27236, 27244, 27245, 27248, 27269, 27506, 27507, 27511, 27513, 27514, 27524, 27535, 27536, 27540, 27756, 27758, 27759, 27766, 27769, 27784, 27792, 277814, 27822, 27826, 27827, 27828, 28415, 28445, 28465, 28485, 28505, 28525, 25491, 27495, 25492, 27495, 25490, 27187, 23490, 1004229, 23491, 24498, 1004546

Prophylactic Fixation:
25491, 27495, 25492, 27495, 25490, 27187, 23490, 1004229, 23491, 24498, 1004546
